# Drug resistant integrase mutants cause aberrant HIV integrations

**DOI:** 10.1186/s12977-016-0305-6

**Published:** 2016-09-29

**Authors:** Janani Varadarajan, Mary Jane McWilliams, Bryan T. Mott, Craig J. Thomas, Steven J. Smith, Stephen H. Hughes

**Affiliations:** 1 HIV Dynamics and Replication Program, Vector Design and Replication Section, National Cancer Institute-Frederick, 1050 Boyles Street, Bldg. 539, Room 130A, Frederick, MD 21702 USA; 2 Division of Preclinical Innovation, National Center for Advancing Translational Sciences, National Institutes of Health, Rockville, MD USA; 3 Department of Pathology, Microbiology and Immunology, Vanderbilt University School of Medicine, Nashville, TN 37232 USA

**Keywords:** Elvitegravir, Aberrant integration, Drug resistance, HIV

## Abstract

**Background:**

HIV-1 integrase is the target for three FDA-approved drugs, raltegravir, elvitegravir, and dolutegravir. All three drugs bind at the active site of integrase and block the strand transfer step of integration. We previously showed that sub-optimal doses of the anti-HIV drug raltegravir can cause aberrant HIV integrations that are accompanied by a variety of deletions, duplications, insertions and inversions of the adjacent host sequences.

**Results:**

We show here that a second drug, elvitegravir, also causes similar aberrant integrations. More importantly, we show that at least two of the three clinically relevant drug resistant integrase mutants we tested, N155H and G140S/Q148H, which reduce the enzymatic activity of integrase, can cause the same sorts of aberrant integrations, even in the absence of drugs. In addition, these drug resistant mutants have an elevated IC_50_ for anti-integrase drugs, and concentrations of the drugs that would be optimal against the WT virus are suboptimal for the mutants.

**Conclusions:**

We previously showed that suboptimal doses of a drug that binds to the HIV enzyme integrase and blocks the integration of a DNA copy of the viral genome into host DNA can cause aberrant integrations that involve rearrangements of the host DNA. We show here that suboptimal doses of a second anti-integrase drug can cause similar aberrant integrations. We also show that drug-resistance mutations in HIV integrase can also cause aberrant integrations, even in the absence of an anti-integrase drug. HIV DNA integrations in the oncogenes BACH2 and MKL2 that do not involve rearrangements of the viral or host DNA can stimulate the proliferation of infected cells. Based on what is known about the association of DNA rearrangements and the activation of oncogenes in human tumors, it is possible that some of the deletions, duplications, insertions, and inversions of the host DNA that accompany aberrant HIV DNA integrations could increase the chances that HIV integrations could lead to the development of a tumor.

**Electronic supplementary material:**

The online version of this article (doi:10.1186/s12977-016-0305-6) contains supplementary material, which is available to authorized users.

## Background

Integration of a DNA copy of the genome of the virus into a host chromosome is an essential step in the retroviral life cycle. Retroviral DNA integration is a two-step process, which begins after the viral enzyme reverse transcriptase synthesizes a DNA copy of the viral genome. In the first step, termed 3′ processing, the virally encoded enzyme integrase (IN) binds to and removes two nucleotides from the 3′ ends of the linear viral DNA. The INs that are bound at the two ends of the viral DNA interact and the resulting IN tetramer (or octamer) holds the 3′ ends of the viral DNA near each other so that they come into close contact with the host DNA. In the second step, the strand transfer reaction, IN promotes an exchange reaction that inserts the newly exposed hydroxyl groups at the 3′ ends of the viral DNA into the two strands of host genome a few nucleotides apart. Host enzymes then repair the nicks in the host genome that are created by the insertion of the viral DNA, creating an integrated provirus. The positions at which these exchange reactions occur on the host DNA generates, after the nicks are repaired, a short duplication of the host sequences that flank the provirus. The length of the duplication in the host DNA depends on the geometry of the IN tetramer/octamer that inserts the two viral DNA ends and is characteristic of the type of retrovirus. The length varies from 4 to 6 base pairs (bp); HIV integration creates 5 bp duplications [1–5].

IN inhibitors are the newest family of anti-HIV drugs to be approved for human use [6]. All of the approved anti-IN drugs preferentially block the strand-transfer step of integration, and the drugs are, for that reason, called IN strand-transfer inhibitors, or INSTIs. INSTIs interact with both IN and the viral DNA substrate and chelate the two metal ions at the IN active site. Upon binding, INSTIs displace the dA at the very 3′ ends of viral DNA and have a π-stacking interaction with the penultimate nucleotide (dC) at both of the 3′ ends of the viral DNA [7–9].

We previously showed that suboptimal doses of the INSTI raltegravir (RAL) can lead to the generation of aberrant proviruses [10]. Generally speaking, these aberrant HIV integrations are quite similar to the aberrant integrations that arose in experiments done with avian sarcoma leukoisis virus (ASLV) vectors. In the ASLV experiments, one of the ends of the viral DNA was mutated in a way that caused it to be a poor substrate for IN-mediated integration [11, 12]. The “good” end of the viral DNA was integrated normally, by IN, whereas the second (mutant) end was apparently inserted by host enzymes. The host-mediated integration events led to the generation of rearrangements of the host DNA, including insertions, deletions, and more rarely, inversions, or in some cases, the transfer of sequences from one chromosome to another. The host-mediated insertions can also lead to deletions or insertions of viral DNA sequences at the 3′ ends of the proviral DNA. In experiments done with HIV-1, we showed that, if the concentration of RAL is suboptimal, it would, in some cases, block the IN-mediated integration of one of the two ends of the viral DNA, causing aberrant integrations similar to those that arise when one end of the ASLV DNA was mutated.

HIV-1 DNA integration into certain host genes can cause the infected cells to divide and persist in patients [13, 14]. The fact that suboptimal doses of RAL can cause HIV integrations that led to significant rearrangements of the host genome, including the transfer of sequences from one chromosome to another, raises the concern that aberrant integrations that involve rearrangements of the host sequences might increase the odds of proliferation of an infected cell more than normal integrations [10]. For that reason, we tested whether other perturbations can cause similar aberrant integrations.

We show here that a suboptimal dose of a second INSTI that has been approved for use in humans, elvitegravir (EVG), can cause aberrant HIV-1 DNA integrations that are quite similar to the aberrant integrations caused by suboptimal doses of RAL. In addition, all anti-HIV drugs can select for resistance; INSTIs are not exceptional in this regard. Because INSTIs bind to the IN active site, the mutations that confer resistance to INSTIs are in or near the active site [8]. Generally speaking, any mutation in an HIV-encoded protein is likely to have some negative impact on the ability of the virus to replicate. However, the regions in and around the active sites of the virally encoded enzymes are particularly well conserved, and it is not surprising that amino acid substitutions in or near the active sites, including drug-resistance mutations, are often deleterious. Some of the INSTI-resistance mutations that are selected because they confer resistance to INSTIs are known to have a considerable negative impact on the ability of IN to integrate viral DNA, and on viral titer. Drug-induced selection for mutations that significantly impact the fitness of the virus are generally considered beneficial for patients; however, that may not always be the case.

We considered the possibility that some of the INSTI-resistance mutations could mimic the effects of a sub-optimal dose of either RAL or EVG to the extent that they might reduce the ability of IN to insert the ends of the viral DNA efficiently. Thus, the reduction in the enzymatic activity of IN caused by these mutations might be sufficient, by itself, to lead to the generation of aberrant proviruses, even in the absence of an added drug. We tested the effects of three IN mutations, Y143R, N155H, and the double mutation G140S/Q148H, all of which reduce the susceptibility of HIV-1 to RAL [15], on viral DNA integration. Two of these mutants, N155H and G140S/Q148H, also had, relative to WT HIV-1, a reduced susceptibility to EVG, and to other INSTIs. Viruses that carry these IN mutations replicate less efficiently than WT [16–19] (Unpublished observations). In the absence of RAL, infection with a vector that carries two of these three IN mutants led to a significant increase in aberrant integrations. These aberrant proviruses have structures that are similar to those that arise upon treatment of the WT virus with suboptimal doses of RAL or EVG. As might be expected, the addition of RAL appeared to increase the fraction of aberrant proviruses that arose during infections with all three of the IN mutants, although the differences we saw in the presence of RAL were not statistically significant. These results raise additional concerns. First, even in the absence of an IN inhibitor, mutations in IN that are selected by treatment with an INSTI can reduce IN enzymatic activity, and this can cause aberrant integrations. Second, because IN mutations make the doses of INSTIs that can be administered to patients suboptimal, treating an infection in which the virus carries INSTI-resistance mutations with an INSTI could increase the probability that aberrant HIV integrations will occur.

## Methods

### Plasmid construction

A four-vector system was used to generate the virus stocks. A ClaI–MluI shuttle cassette that contains sequences that permit the replication of circular forms of viral DNA as plasmids in *Escherichia coli* was derived from pHIV-SH [20]. The cassette was shortened by removing the Pol II promoter. The final cassette contained a zeocin resistance gene with an upstream EM-7 Promoter (EM-zeo), a lac operator sequence, and a ColE1 origin of replication (oriE). A coding region for enhanced green fluorescent protein (eGFP), under the control of the cytomegalovirus promoter (CMV), was inserted as a NotI–ClaI fragment immediately upstream of the plasmid-recovery cassette. The plasmid used to produce the viral RNA, pSICO-LZF, was derived from pSICO-XBX by inserting the shuttle cassette and the eGFP coding region as a NotI–MluI fragment [21]. The viral RNA was expressed from a chimeric 5′ LTR with a CMV promoter in place of U3, and the vector also contained an HIV Psi packaging sequence and a Rev Response element (RRE)/RNA export signal [22]. The four-vector system included, in addition to pSICO-LZF, a plasmid expressing HIV-1 Gag and Gag–Pol: pMDL-SH.IN+; a plasmid that expresses REV: pRSV-REV; and a plasmid that expresses VSV-G: pCMV-VSV-G. pMDL-SH.IN+ was derived from pMDLg/pRRE by replacing the *gag* and *pol* genes with the equivalent sequences from pHIV-SH [20]. Both the pRSV-REV and the parental pMDLg/pRRE expression plasmids were obtained from Didier Trono (École Polytechnique Fédérale de Lausanne, Lausanne, Switzerland) through Addgene, Inc. [22]. The three IN mutants used in the experiments have been described [15]. The segments carrying the mutant forms of IN were moved into the pMDL-SH.IN+ plasmid by transferring the segment of the Gag–Pol coding sequence between PstI and AflII from pNLNgoMIVR-ΔEnv.Luc into pMDL-SH.IN+ using the corresponding restriction enzyme sites.

### Cells, transfection, and infection

Human embryonic kidney (HEK) 293T and human osteosarcoma (HOS) cells were maintained in DMEM (Cellgro) supplemented with 5 % (vol/vol) FBS, 5 % (vol/vol) newborn calf serum, 100 U/mL penicillin G, and 100 µg/mL streptomycin (Quality Biological). Recombinant virus stocks were generated by calcium phosphate-mediated cotransfection of 293T cells seeded at 1.5 × 10^6^ cells in 100-mm culture plates with the plasmids that make up the four-vector system. Thirteen micrograms of pSICO-LZF (or pSICO-LZR), 12 µg pMDL-SH.IN+, 5 µg pRSV-REV, and 4 µg pCMV-VSV-G were used per plate. 6 h after transfection, cells were gently washed three times with PBS, and fresh media was added. Virus-containing supernatants were harvested 48 h after transfection, clarified by centrifugation at 1620×*g* for 10 min, and incubated at 37 °C with 500 U of DNase I (Invitrogen) per 50 mL virus in 5 mM MgCl_2_ to remove any residual vector DNA carried over from the transfection. HOS cells were seeded at 7.5 × 10^5^ per 100-mm plate the day before infection. Peripheral blood mononuclear cells (PBMCs) from healthy human donors were seeded at a minimum of 5 × 10^6^ cells per 25-cm^2^ cell culture flask (Corning) and maintained in RPMI media supplemented with 10 % FBS, 100 U/mL penicillin G, 100 µg/mL streptomycin, 2 mM l-glutamine, and 50 U IL-2. The PBMCs were activated with 5 µg/mL phytohemagglutinin-P (PHA-P) for 48–72 h. The cells were then collected by centrifugation and resuspended in fresh media containing IL-2. Drug-free cells were infected with 50 ng/mL p24 recombinant virus in the presence of 4 µg/mL of polybrene (Sigma-Aldrich). The cells that were treated with various suboptimal doses of RAL (a gift from Daria Hazuda, Merck Research Laboratories, West Point, PA) or EVG [23] were preincubated with the drug for 3 h at 37 °C and then infected with 50 ng/mL p24 recombinant virus in the presence of 4 µg/mL of polybrene. The appropriate concentration of RAL or EVG was maintained during the infection. After 24 h of incubation with the virus, the virus-containing media was removed and replaced with fresh media (without virus) in the drug-free cells, whereas fresh media (without virus) containing the appropriate concentrations of RAL or EVG was added to the drug-treated cells. The cells were harvested 4 days after infection, and DNA was extracted using the QIAmp DNA Blood Kit from Qiagen.

### Recovery of integrated retroviral DNA

Genomic DNAs (100 µg) isolated from the infected HOS cells or PBMCs were treated with DpnI for 2 h at 37 °C to eliminate any remaining plasmid DNA that might have been carried over from the transfection. The DpnI-digested DNA was heated at 80 °C for 20 min and then ethanol precipitated. The DNA was digested overnight with BclI at 50 °C, extracted with phenol/chloroform, and ethanol precipitated. DNA was resuspended in 890 µL of nuclease-free water and was self-ligated overnight at 16 °C in the presence of T4 DNA Ligase and ligation buffer (NEB) in a final volume of 1 mL. The mix was ethanol precipitated and resuspended in 100 µL of nuclease-free water.

DNA (up to 700 ng) was introduced into ElectroMAX DH10B *E. coli* cells by electroporation using the BTX Electroporation System at 186 Ω and 2.5-kV resistance. The bacterial cells were allowed to recover in 500 µL supra optimal broth with catabolite repression (SOC) media for 1.5 h at 37 °C with shaking, and plated on L-broth plates containing 100 µg/mL zeocin. The next day, colonies were picked and grown overnight in L-broth containing 100 µg/mL zeocin. DNA was purified using the Qiagen BIO ROBOT Universal System.

### Sequencing of the integrated viral DNA

Recovered plasmids were directly sequenced using the primers LTR-FOR (5′ GACTTACAAGGCAGCTGTAG), which hybridizes to the vector genome in a region that just precedes the poly purine tract (PPT), and pSICO REV (5′ GCCTCTTGCCGTGCGCGCTTC), which hybridizes near the primer binding site (PBS), between the LTR and *gag*. All sequencing was performed by Macrogen (Rockville, MD). In some cases the proviruses had sustained such large deletions that it was necessary to use additional primers to do additional sequencing to determine the end of the provirus. Human and viral sequences were analyzed by BLAST.

## Results

### Suboptimal doses of elvitegravir (EVG) can cause aberrant integration of HIV-1 DNA

We previously showed that infecting cells in the presence of suboptimal doses of RAL can cause aberrant HIV-1 integrations, and we proposed that this was the result of the drug blocking the IN-mediated insertion of only one of the two ends of the viral DNA. If that view is correct, a suboptimal dose of any INSTI should cause similar aberrant integrations. Broadly speaking, the results we obtained with suboptimal doses of EVG were quite similar to the results we previously obtained with RAL. In both sets of experiments, we used an HIV-based viral vector that was generated using a 4-plasmid system (see “Methods” section and Additional file 1: Figure S1). There are advantages in using a one-round vector to do the experiments we report here. First, one-round vectors are safer than replication-competent HIV-1, particularly if drug-resistant mutants are being used in the experiments. Second, if a one round vector is used, it is relatively easy to prepare stocks that have a similar amount of infectious virus present. More importantly, if the experiments involve mutant viruses that grow at different rates, differential replication will not affect the relative amounts of the viruses that are present throughout the experiment. Third, there can be no question that the virus could have reverted during the course of the experiment. Fourth, it is much easier to accurately measure the impact of a given dose of an inhibitor in an experiment that uses single-round as opposed to a multi-round vectors. In addition, integration of the vector DNA is carried out by normally by IN, and we showed that the replication of wild-type and drug-resistant versions of these vectors respond accurately and appropriately to a variety of INSTIs, including RAL, EVG, and dolutegravir (DTG) [9, 15, 24–28]. Finally, we previously used these vectors to show that RAL can cause aberrant integrations [10].

Viral DNAs were recovered from HOS cells infected with the HIV vector in the presence of suboptimal doses of EVG and sequenced with HIV-1 specific primers as described previously [10]. Before we began doing experiments perturbing HIV integration, it was known that normal HIV-1 integration produces proviruses that have each of the LTRs inserted into the host genome with a loss of 2 bp from each end, and that the integrated proviruses are flanked by a 5 bp repeat of the host DNA. To show that we could reproduce these results using our vector system, we previously showed that all 99 of the proviruses we recovered from an unperturbed infection of HOS cells with the WT vector were normal [10]. We got similar results when we infected PBMCs; none of the 85 proviruses we isolated were aberrant. We have used the data from these WT vector controls to calculate the statistical significance of the integration site data we present here.

As we previously reported for suboptimal doses of RAL, the addition of suboptimal doses of EVG led to the recovery of aberrant proviruses when the infections were done with a vector that carried WT IN (Table 1). We also recovered, in addition to the normal and aberrant proviruses, unintegrated circular viral DNAs, 1- and 2-LTR circles and auto-integrants in the experiments done with the WT IN vector in the presence of sub-optimal doses of the EVG (Table 1). Some of these circular forms of the viral DNA had aberrant structures; the numbers of the normal and aberrant circular forms recovered in each of the experiments are given in the tables. As expected, the ratio of the unintegrated forms of the viral DNA to the integrated forms went up when the infections were done in the presence of EVG. These results are similar to the results we obtained with sub-optimal RAL (10).

**Table 1:** Recovery of integrated viral DNA from elvitegravir treated cells

HOS cells; drug concentration	Aberrant integration	Normal integration	% Aberrant integration	1-LTR (aberrant 1-LTR)	2-LTR (aberrant 2-LTR)	Autointegrants (aberrant circles)	Total no. of samples recovered and sequenced
No drug*	0	99	0	7 (0)	0 (0)	1 (2)	120
IC_14_**	4	39	9.3	108 (21)	19 (5)	10 (56)	288
IC_30_	1	14	6.6	102 (31)	10 (8)	11 (58)	276
IC_50_	2	19	9.5	116 (18)	28 (14)	11 (47)	288
IC_60_	2	19	9.5	125 (11)	26 (10)	12 (52)	288
IC_75_	8	46	14.8	93 (25)	13 (13)	3 (57)	287

* Data for the no drug control are from Varadarajan et al. [10]

** EVG IC_50_: 6.57 nM

We recovered a larger number of proviruses at the highest dose of EVG (IC_75_) than at the lower doses. This suggests that, for some reason, the recovery of the integrated viral DNA was particularly efficient for this sample. Although we strive to do the experiments exactly the same way for each sample, there are steps in the procedure (for example the ligation step which forms circular DNAs from the integrated proviruses) whose efficiency cannot be accurately monitored. Variation in the efficiency of these steps can have a profound effect on the number of the proviruses that are recovered. For that reason, the ratio of aberrant to normal proviruses is a better measure of the impact of the drugs on integration than is a comparison of the absolute number of integrated and unintegrated viral DNAs recovered. In Table 1, the ratio of aberrant to normal proviruses ranged from ~1/15 to ~1/5. With the exception of the data for the IC_30_, where the dataset was small, compared to the total number of samples recovered and sequenced, the data for all of the other EVG concentrations showed (using Fisher’s exact test) a statistically significant difference (p < 0.05) in the fraction of aberrant integrations compared with the no drug control. Although a larger fraction of the proviruses we recovered were aberrant at the higher drug concentrations, the absolute number of aberrant proviruses was relatively small, and the differences seen in the fraction of aberrant integrations at the different drug concentrations used were not statistically significant.

We analyzed the structures of the aberrant proviruses. A description of the defective proviruses isolated in the experiments described in Table 1 is given in Additional file 2: Table S1. In most cases, the aberrant proviruses have one end in which the viral DNA, and the junction to the host genome, appear to be normal, which supports our assumption that this end was appropriately inserted by IN. The aberrant end of the viral DNA was often, but not always, truncated. The aberrant viral DNA end could be joined either to additional viral DNA sequences (in either orientation), or to sequences from the host genome. Most commonly, the host DNA was duplicated where it was joined to the aberrant viral DNA end. However, instead of a short 5 bp repeat, in the aberrant proviruses harboring a duplication of the host sequences, the repeats or duplications were usually much larger, and could be hundreds or thousands of bp long. Alternatively, there were deletions of the flanking host DNA, and less frequently inversions. There were, in some cases, insertions of host sequences from other chromosomes. In terms of their structure, the aberrant proviruses produced by infections done in the presence of suboptimal doses of EVG appeared to be quite similar to the aberrant proviruses produced by infection in the presence of suboptimal doses of RAL. We also recovered circular forms of viral DNA that have host sequences inserted between the LTRs, which do not appear to derive from proviruses. Viral DNAs of this type have been recovered before, in experiments done with mutants of an ASLV-based vector. We proposed that these circular DNAs derived from abortive integration events [29].

### Drug resistance mutations in IN can cause aberrant integrations

If, as we have suggested, a suboptimal dose of an INSTI can block the integration of one end of the viral DNA, that could lead to aberrant integrations. This suggests the possibility that IN mutations that significantly reduce the activity of the enzyme could also cause similar aberrant integrations, even in the absence of an anti-IN drug. We tested three IN mutants, all of which reduced the susceptibility of the virus (and IN) to RAL, and caused a reduction in the relative infectivity of the single-round HIV vector used in our assay system (Y143R, residual titer is 40–45 % of WT; N155H, ~40 % of WT, and G140S/Q148H, 30–35 % of WT). The Y143R mutant specifically causes resistance to RAL; the other two mutants also cause cross-resistance to other INSTIs. We addressed two questions: (1) Would any of these three IN mutants cause aberrant integrations in the absence of an added INSTI?, and (2) Would the addition of a suboptimal dose of an INSTI affect (increase) the propensity of these three IN mutants to make aberrant integrations? Because the drug-resistant mutants are considerably less sensitive to inhibition by RAL, we used much higher concentrations of RAL as a suboptimal dose. The concentrations of RAL were chosen so that the level of infectivity of the mutants was reduced to match two of the decreases in infectivity in the experiments done with the WT vector (IC_50_ and IC_75_). We previously showed that we obtained similar results, in terms of RAL causing aberrant integrations, in a standard cell line (HOS) and in PBMCs [10]. Here, we tested the effects of one of the mutants (N155H) in the absence of any added drug in both HOS cells and PBMCs.

**Fig. 1:**
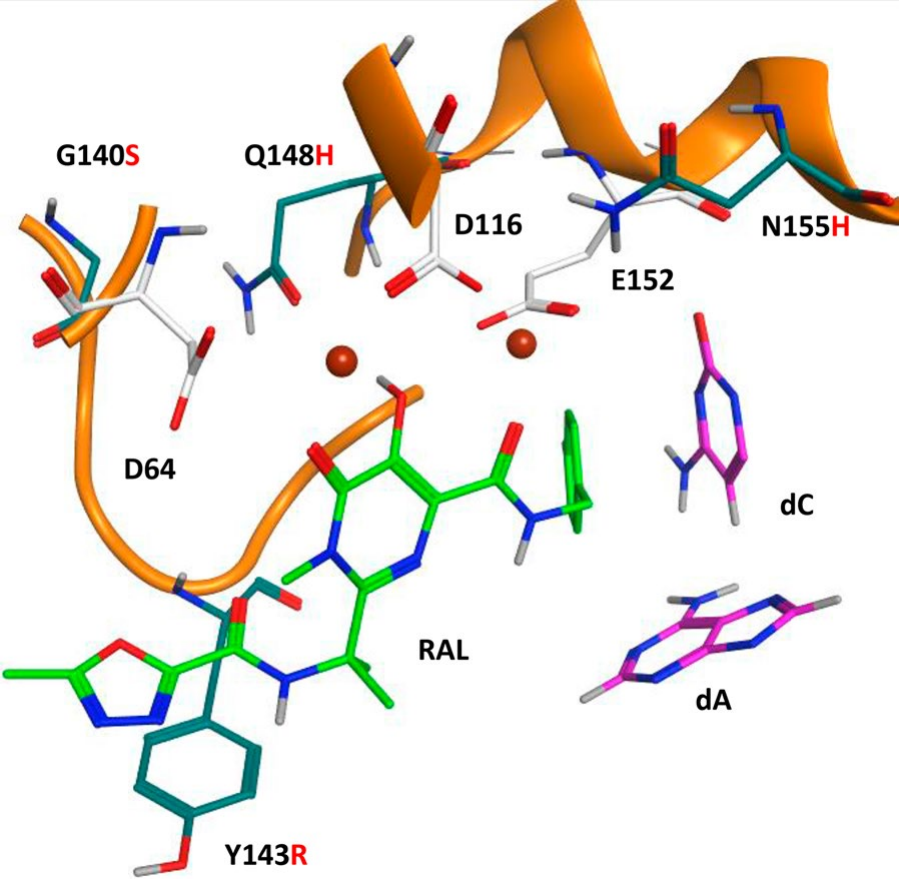
RAL bound in the IN active site. Model showing raltegravir (RAL) bound in the active site of HIV-1 IN (see Hare et al. [9]). The bound form of RAL (*green*) chelates the Mg^2+^ ions at the active site through the diketo motif. The oxidazole ring and benzyl moiety of RAL interact, through π–π stacking, with Y143 (*teal*) and the penultimate cytosine (*magenta*) at the 3′ end of the viral DNA strand, respectively. Binding of RAL causes the adenosine (*magenta*) to move away from IN active site (side chains are *white*), which inhibits the strand transfer reaction. Residues that are mutated in resistant forms of IN are shown (side chains colored teal), and the letter designating the mutant amino acid is indicated in *red*

The Y143R mutation is RAL-specific; Y143 has a π–π interaction with the oxadiazole ring of RAL. This moiety is not present in the other approved INSTIs [30], and, for that reason, Y143R does not confer cross resistance to the other INSTIs. Although position 143 is near the IN active site, it is farther from the metal ions than the other resistance mutations that we tested (positions 140, 148, and 155; see Fig. 1). In the absence of any added RAL, we found aberrant integrations in infections that were done using the Y143R mutant; however, for the Y143R mutation, the ratio of aberrant to normal integrations was relatively low (1/30), and the fraction of aberrant integrations was not, when compared to infections done with WT IN in the absence of added RAL, statistically significant. This ratio increased in the presence of a suboptimal dose of RAL (~1/8 at the IC_50_ and ~1/10 at the IC_75_) (Table 2; Additional file 2: Table S2), and the fraction of aberrant proviruses obtained at both the IC_50_ and IC_75_ were significant (p < 0.006 and p < 0.009, respectively), when compared to the control done with WT IN in the absence of any added drug. This is important because the Y143R mutation raised the IC_50_ for RAL substantially, (IC_50_ 425 nM, see Table 2), and doses of RAL that would be optimal for WT would be suboptimal for the Y143R mutant. A description of the defective proviruses isolated in the experiments described in Table 2 is given in Additional file 2: Table S2. In all of these experiments, the number of aberrant integrations was small. Although the data suggest that a suboptimal concentration of RAL appears to increase the propensity of the Y143R mutant to make aberrant integrations, the differences in the fraction of integration sites that were aberrant for the Y143R mutant, comparing the presence and absence of RAL, were not statistically significant.

**Table 2:** Recovery of integrated viral DNA from cells infected with the IN mutant Y143R

HOS cells, drug concentration	Aberrant integration	Normal integration	% Aberrant integration	1-LTR (aberrant 1-LTR)	2-LTR (aberrant 2-LTR)	Autointegrants (aberrant circles)	Total no. of samples recovered and sequenced
No drug	2	62	3.1	94 (19)	12 (9)	9 (55)	288
RAL IC_50_	4	35	10.2	87 (7)	23 (18)	8 (75)	288
RAL IC_75_	4	42	8.7	83 (17)	40 (11)	4 (61)	288

RAL IC_50_: 425 nM

The N155H mutation lies near the sites where the metal ions are bound in the IN active site, and near the 3′ end of the viral DNA. In the absence of any added RAL, the ratio of aberrant to normal integrations is about 1 to ~7 in infected HOS cells (Table 3); this difference is statistically significant (p = 0.002). A description of the defective proviruses isolated in the experiments described in Table 3 is given in Additional file 2: Table S3. Adding RAL at the IC_50_ concentration caused an increase to ~1/4. There may have been a modest increase in the fraction of aberrant proviruses at IC_75_ (~1/3); however, because the sample size is small, this apparent difference was not statistically significant. To be sure that the IN mutants would also cause similar aberrant integrations in PBMCs, we infected these cells with the N155H mutant. The ratio of aberrant to normal integrations appeared to be lower in the absence of any added drug in the PBMCs (~1/10). When these data were compared to data from a control experiment in which PBMCs were infected with the WT virus (10), the difference was statistically significant (p = 0.006). Although the ratio of aberrant to normal integrations appeared to be slightly lower in PBMCs than in HOS cells (~1/7), this difference was not statistically significant.

**Table 3:** Recovery of integrated viral DNA from cells infected with the IN mutant N155H

Cell type, drug concentration	Aberrant integration	Normal integration	% Aberrant integration	1-LTR (aberrant 1-LTR)	2-LTR (aberrant 2-LTR)	Autointegrants (aberrant circles)	Total no. of samples recovered and sequenced
HOS							
No drug	5	36	12.2	82 (8)	27 (17)	4 (66)	287
RAL IC_50_	6	26	18.7	75 (15)	55 (23)	6 (43)	288
RAL IC_75_	2	6	25	93 (7)	57 (22)	5 (55)	288
PBMC							
No drug	10	103	8.8	82 (4)	15 (1)	0 (36)	285

RAL IC_50_: 297 nM

The Q148H mutation is also near the two metal ions at the IN active site, and the 3′ end of the viral DNA. The G140S mutation is adjacent to Q148H (Fig. 1). In the absence of any added drug, this double mutation also caused aberrant mutations; the ratio of aberrant to normal mutations in HOS cells was ~1/11 (Table 4); this is significant compared to the no drug control (p = 0.007). A description of the defective proviruses isolated in the experiments described in Table 4 is given in Additional file 2: Table S4. The ratio was similar at the IC_50_ (~1/13), and the ratio appeared to increase to ~1/6 at the IC_75_; again, the numbers of aberrant integrations are relatively small, and the differences seen for this mutant in the presence and the absence of added drug were not statistically significant. As expected, we also recovered unintegrated circular viral DNAs when cells were infected with viruses that carried mutant forms of IN (Tables 2, 3, 4).

**Table 4:** Recovery of integrated viral DNA from cells infected with the IN mutant G140S/Q148H

HOS cells, drug concentration	Aberrant integration	Normal integration	% Aberrant integration	1-LTR (aberrant 1-LTR)	2-LTR (aberrant 2-LTR)	Autointegrants (aberrant circles)	Total no. of samples recovered and sequenced
No drug	5	56	8.2	72 (17)	25 (5)	7 (50)	288
RAL IC_50_	5	66	7	38 (6)	32 (15)	9 (85)	288
RAL IC_75_	7	45	13.5	56 (10)	38 (13)	6 (72)	288

RAL IC_50_: 4.25 µM

## Discussion

It appears to be relatively easy to (partially) disrupt the HIV-1 integration process in ways that produce aberrant integrations. If the integration reaction is completely blocked, viral DNA is almost never inserted into the host genome. However, as has already been discussed, it is possible to create conditions under which IN can insert only one end of the viral DNA. When this happens, host enzymes insert the second viral DNA end into the host genome with an efficiency that can be as high as 30 % of the rate at which a normal viral DNA end can be inserted by IN [12]. This produces what at first appears to be a complex array of host/virus DNA junctions, in which the viral DNA sequences are often (but not always) truncated. The host and virus DNAs at these aberrant junctions can be duplicated, deleted, inverted, and sequences from other chromosomes can be brought in.

However, the data suggest that there is a single underlying theme, and that a relatively simple set of processes, which can be catalyzed by well-characterized host machinery, can account for all of the aberrant proviruses: (1) The unintegrated 3′ end of the viral DNA is used by a host DNA polymerase to prime DNA synthesis; nearby DNA sequences (either host or viral) are used as the template, and (2) The host polymerase creates an extended DNA (which is attached to the 3′ end of the viral DNA) that has homology to nearby host DNA. Homologous recombination creates aberrant proviruses that, collectively, correspond to the structures of the aberrant proviruses we have mapped.

First, the 3′ end of the viral DNA is used to prime DNA synthesis. Because integration takes place in the nucleus of the infected cell, there are a number of host DNA polymerases present that are involved in the repair of the host genome [31–33]. Because these host DNA polymerases cannot initiate DNA synthesis de novo, they require, in addition to the viral DNA, which is used as the primer, a template. This explains the fact that, at the aberrant end of the viral DNA there is usually, although not always, a short segment that is homologous to the appended DNA sequence. In the search for a homologous template sequence on which to prime, the viral DNA end can be truncated by the host DNA polymerase (or an ancillary factor). Although it is common for the host DNA polymerase that is extending the 3′ end of the viral DNA to copy an adjacent host sequence, it is also possible for the polymerase to copy a segment from either strand of the viral DNA. Thus the polymerization reaction appends whatever host or viral DNA sequence is copied onto the 3′ end of the viral DNA. This process is not confined to copying a single viral or host template, and multiple sequences can be appended to the 3′ end of the viral DNA. For the viral DNA to be successfully integrated, the polymerization reaction must copy sequences on the chromosome in which IN made the initial insertion. Once a segment near the original integration site has been copied, homologous recombination can join the aberrant DNA end to the host genome. It should be clear that this homologous recombination can take place on either side of the initial IN-mediated event. Thus the host-mediated insertion reaction can lead to a deletion in the flanking host DNA if it takes place on one side of the initial IN-mediated integration event; if it takes place on the other side, it will cause a duplication of the flanking host DNA, as was shown in experiments done using ASLV-based vectors [11, 12, 34].

Although we have now found three different sets of circumstances that can cause a similar set of aberrant integrations, here too, there is a single, straightforward, underlying theme. In the initial experiments, done using an ASLV vector, we made mutations in the viral genome that led to the synthesis of linear viral DNAs that had one end that was not a good substrate for IN. To our surprise, this had, in some cases, only a modest impact on the titer [12]. As was discussed earlier, we showed, by isolating the resulting proviruses that, if one end of the viral DNA was inserted into the host genome by IN, the other could be efficiently inserted into the genome by the host machinery. In thinking about the effects of a suboptimal dose of an INSTI, we thought it possible that a suboptimal dose of an INSTI could mimic the effects of a viral DNA with a bad end: one end of the viral DNA could be inserted by IN, the other (blocked by the INSTI) would be inserted by the host machinery. We previously showed that suboptimal doses of RAL could produce aberrant integrations that are similar to those produced by making viral DNAs with one bad end. We now show that EVG has the same effect, and it seems very likely that a suboptimal dose of any INSTI will produce similar results.

In both of these scenarios, there is a considerable difference in the ability of IN to insert the two different ends of the viral DNA; one end will be inserted with the normal high efficiency, the other very poorly, or not at all. For that reason we were curious about the effect of reducing the overall activity of IN on aberrant integrations. In this case there would be a similar reduction in the ability of IN to insert each of the two ends of the viral DNA. Experiments done using some of the common RAL-resistant mutants, which are known to reduce the activity of IN, produced aberrant integrations in the absence of any added INSTI. These aberrant integrations appear to be very similar to those produced either by using a viral DNA with a bad end, or by using a suboptimal dose of an INSTI. This suggests that there is a relatively modest excess of IN enzymatic activity, relative to what is needed to carry out the integration of the two ends of the viral DNA, and that there is a limited time during which the integration reaction can take place.

One of the consequences of having an IN that carries resistance mutations is that these mutations raise the IC_50_ for the drug, making drug concentrations that are optimal against the WT virus suboptimal for the resistant mutants. We show here that, although the mutations that confer resistance to RAL are capable of causing aberrant integrations in the absence of an added INSTI (RAL in our case), the addition of high levels of RAL appeared to increase the ratio of aberrant to normal integrations, although the numbers of aberrant integrations we recovered was small, and the differences were not statistically significant. This means that, in a patient who is undergoing treatment with an INSTI, the drug resistance mutations would have two paths by which they would cause aberrant mutations (1) by affecting the enzymatic activity of IN and (2) by causing what would normally be an optimal dose of the INSTI to be suboptimal.

It has long been thought that HIV proviruses, in contrast to the proviruses of a number of other retroviruses, do not integrate into places in the genome that can stimulate the growth the infected cells. However, both we and others recently showed that HIV integration into certain oncogenes (BACH2 and MKL2) can lead to the clonal expansion of the infected cells [13, 14]. In a mouse model, activation of the BACH2 gene by the integration of an MLV provirus can cause tumors [35]. Although a direct link between HIV integration and oncogenic transformation has not been established, there is, in the literature, a report of a tumor which has an HIV integration in BACH2 [36]. Because there is only this one report, its significance is not yet clear. However, the available data suggest the possibility that HIV integration could potentially lead to tumorigenesis. As we previously pointed out, the fact that the aberrant integrations that are caused by suboptimal doses of an INSTI, or, as we report here, by INSTI-resistance mutations even in the absence of an INSTI, is cause for concern. This concern is based on the observation that the aberrant integrations can involve substantial rearrangements of the host genome, including duplications, deletions, inversions, and the insertion of sequences from other chromosomes. DNA rearrangements in both BACH2 and MKL2 are known to play a role in human cancers [37–40]. If normal HIV integrations in BACH2 and MKL2 can cause the clonal expansion of the infected cells, it would seem that the sorts of aberrant integrations we have described are more likely to alter the expression of genes than are normal integrations, which could have unwanted, and undesirable, consequences.

## Conclusions

We previously showed that suboptimal doses of an IN inhibitor RAL caused aberrant integrations that included deletions, duplications, and transfer of genetic information from one chromosome to another. We show here that a second IN inhibitor, EVG, can cause similar aberrant integration. More importantly, we also show that some IN inhibitor resistance mutations that reduce the enzymatic activity of IN can cause aberrant integrations, even in the absence of an inhibitor. At least two of the three clinically relevant drug resistant integrase mutants we tested, N155H and G140S/Q148H, which reduce the enzymatic activity of integrase, caused aberrant integrations, even in the absence of any added drug. In addition, these drug resistant mutants increase the IC_50_ for anti-integrase drugs, and concentrations of the drugs that would be optimal against the WT virus are suboptimal for the mutants. Normal HIV integrations into the oncogenes BACH2 and MKL2 can cause the clonal expansion and proliferation of the infected cells, and rearrangements of BACH2 and MKL2 can cause cancer in humans. For these reasons, we suggest that there should be concern about therapies, and mutations in HIV, that can lead to aberrant integrations that involve deletions, duplications and rearrangements of the host DNA.

## Additional files

10.1186/s12977-016-0305-6 Structure of DNAs used to generate the HIV-1 vectors. At the top is a diagram of the DNA used to generate the genomic RNA that is packaged into the viral vector. When the vector infects human cells, a DNA copy of vector genome is inserted into host DNA. This allows the expression of GFP, which is under the control of a CMV promoter. The vector genome also carries an *E. coli* plasmid origin of replication (Ori) and a zeocin resistance gene that allows circular DNA forms of the vector genome to replicate and be selected in *E. coli*. The other three DNAs express Gag–Pol, Rev, and VSV-G. Rev is expressed from an RSV promoter; Gag–Pol and VSVG are expressed from CMV promoters. All four DNAs are grown in *E. coli* as plasmids. The plasmids encode ampicillin resistance (Ampr).
10.1186/s12977-016-0305-6 Structure of the aberrant proviruses isolated from cells treated with suboptimal concentrations of EVG (Table S1); from cells infected with viruses harboring RAL resistant mutations and treated with suboptimal doses of RAL (Tables S2–S4). The inhibitory concentrations of EVG or RAL used to treat the cells are indicated. The aberrations in the viral LTRs and in the integrated host chromosome(s) are indicated. U-LTR: the LTR adjacent to the primer binding site; D-LTR: the LTR adjacent to the polypurine tract.
