# Supplementary material for: Drug resistant integrase mutants cause aberrant HIV integrations
Source: Retrovirology. 2016 Sep 29;13:71. doi: 10.1186/s12977-016-0305-6 (PMC5041404; doi:10.1186/s12977-016-0305-6)
Supplement: Supplementary file 1 — 10.1186/s12977-016-0305-6 Structure of DNAs used to generate the HIV-1 vectors. At the top is a diagram of the DNA used to generate the genomic RNA that is packaged into the viral vector. When the vector infects human cells, a DNA copy of vector genome is inserted into host DNA. This allows the expression of GFP, which is under the control of a CMV promoter. The vector genome also carries an E. coli plasmid origin of replication (Ori) and a zeocin resistance gene that allows circular DNA forms of the vector genome to replicate and be selected in E. coli. The other three DNAs express Gag–Pol, Rev, and VSV-G. Rev is expressed from an RSV promoter; Gag–Pol and VSVG are expressed from CMV promoters. All four DNAs are grown in E. coli as plasmids. The plasmids encode ampicillin resistance (Ampr). [file 12977_2016_305_MOESM1_ESM.pptx]

## Slide 1
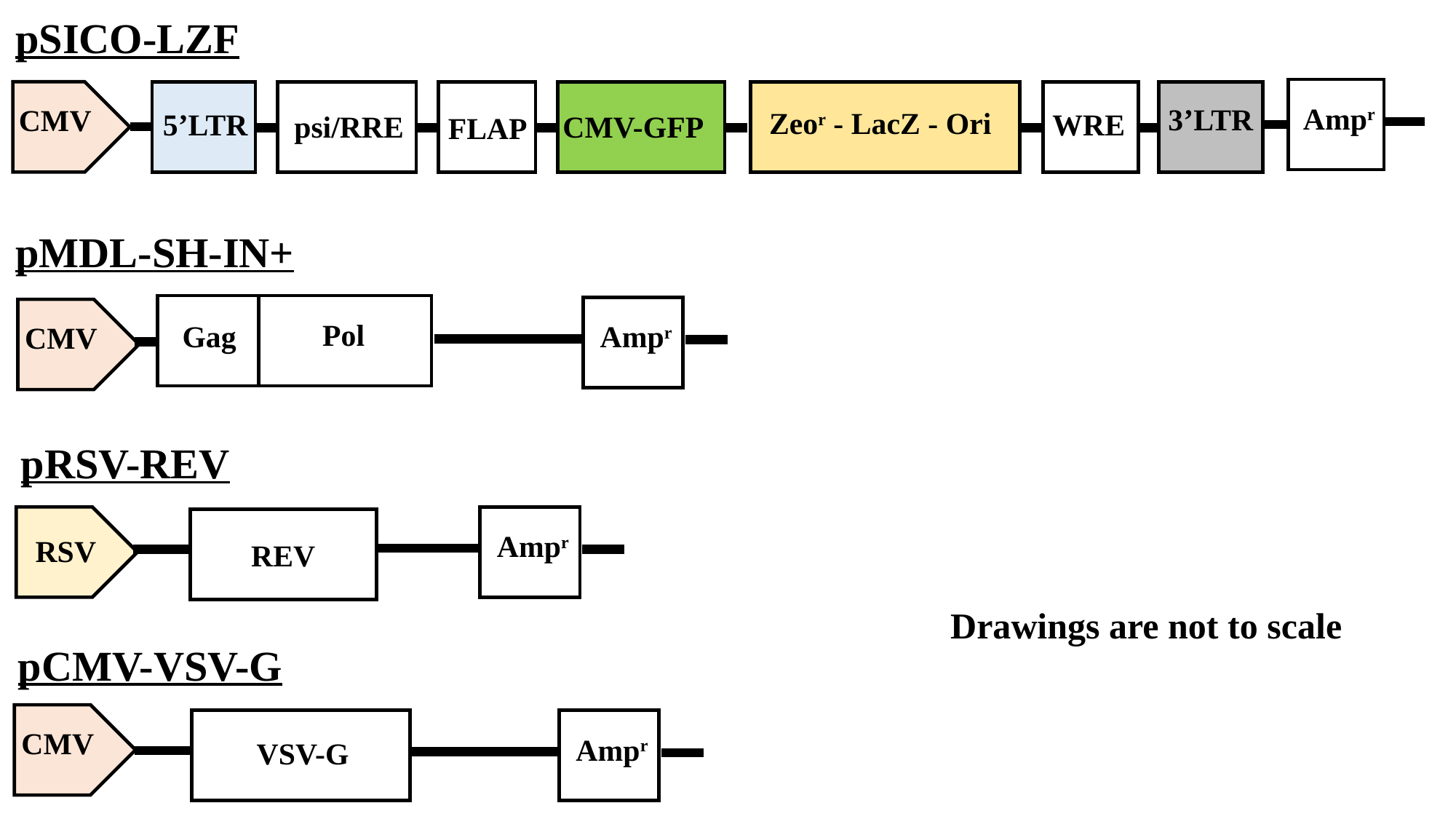

pSICO-LZF
Ampr
3’LTR
CMV
Zeor - LacZ - Ori
5’LTR
WRE
psi/RRE
CMV-GFP
FLAP
pMDL-SH-IN+
Pol
Gag
Ampr
CMV
pRSV-REV
Ampr
RSV
REV
Drawings are not to scale
pCMV-VSV-G
CMV
Ampr
VSV-G
