# Supplementary material for: Drug resistant integrase mutants cause aberrant HIV integrations
Source: Retrovirology. 2016 Sep 29;13:71. doi: 10.1186/s12977-016-0305-6 (PMC5041404; doi:10.1186/s12977-016-0305-6)
Supplement: Supplementary file 2 — 10.1186/s12977-016-0305-6 Structure of the aberrant proviruses isolated from cells treated with suboptimal concentrations of EVG (Table S1); from cells infected with viruses harboring RAL resistant mutations and treated with suboptimal doses of RAL (Tables S2–S4). The inhibitory concentrations of EVG or RAL used to treat the cells are indicated. The aberrations in the viral LTRs and in the integrated host chromosome(s) are indicated. U-LTR: the LTR adjacent to the primer binding site; D-LTR: the LTR adjacent to the polypurine tract. [file 12977_2016_305_MOESM2_ESM.docx]

**Structure of the aberrant proviruses**

**Supplementary Table 1: Elvitegravir**

|  | U-LTR | D-LTR | Chromosome | Chr # |
| --- | --- | --- | --- | --- |
| IC_14_ |  |  |  |  |
| 1 | 6 bp Deletion | Normal | Duplication - Bcl1 site within the duplication | Chr 1 |
| 2 | Entire LTR deleted | Normal | Duplication - 1 bp | Chr 2 |
| 3 | 400 bp deletion | 300 bp deletion | Insertion of DNA from a different chromosome | Chr 6 & 9 |
| 4 | Entire LTR deleted | Normal | Circular viral DNA carrying 283 bp host sequence between the proviral ends | Chr 22 |
|  |  |  |  |  |
| IC_30_ |  |  |  |  |
| 1 | Entire LTR deleted | 400 bp deletion | Insertion of DNA from a different chromosome | Chr 2 & 9 |
|  |  |  |  |  |
| IC_50_ |  |  |  |  |
| 1 | Normal | Normal | Deletion - 67 Kb | Chr 18 |
| 2 | Entire LTR deleted | 60 bp insertion at proviral DNA end | Duplication ~ 440 bp | Chr 11 |
|  |  |  |  |  |
| IC_60_ |  |  |  |  |
| 1 | Normal | 1 bp deletion | Duplication - 4 bp | Chr 14 |
| 2 | Entire LTR deleted | Normal | Circular viral DNA carrying 294 bp host sequence between the proviral DNA ends | Chr 12 |
|  |  |  |  |  |
| IC_75_ |  |  |  |  |
| 1 | Normal | 1 bp insertion | Duplication - 178 bp | Chr 6 |
| 2 | 2 bp insertion | Normal | Duplication - 1 bp | Chr 15 |
| 3 | Entire LTR deleted | Normal | Circular viral DNA carrying 684 bp host sequencce between the proviral DNA ends | Chr 3 |
| 4 | Normal | 2 bp deletion | Duplication 3 bp | Chr 8 |
| 5 | Normal | Entire LTR deleted | Duplication - 6 bp | Chr 4 |
| 6 | Entire LTR deleted | 6 bp deletion | Insertion of different fragments from Chr 20 and duplication of the sequences | Chr 20 |
| 7 | 350 bp deletion | Entire LTR deleted | Insertion of DNA from a different chromosome | Chr 1 & 20 |
| 8 | Normal | 3 bp deletion | Duplication ~ 930 bases | Chr 6 |

**Supplementary Table 2: Raltegravir resistant mutant Y143R**

|  | U-LTR | D-LTR | Chromosome | Chr # |
| --- | --- | --- | --- | --- |
| No Drug |  |  |  |  |
| 1 | 6 bp deletion | Normal | 0 bp duplication | Chr 11 |
| 2 | Entire LTR deleted | 220 bp deletion | Inversion of the Chromosome | Chr 1 |
|  |  |  |  |  |
| IC_50_ |  |  |  |  |
| 1 | Normal | Normal | Duplication 4 bp | Chr 2 |
| 2 | Normal | Normal | Duplication 4 bp | Chr 5 |
| 3 | 19 bp deletion | Normal | Insertion of different chromosome | Chr 22 & 10 |
| 4 | Normal | Normal | Circular viral DNA carrying 366 bp of host sequence between the proviral DNA ends | Chr 5 |
|  |  |  |  |  |
| IC_75_ |  |  |  |  |
| 1 | Normal | 10 bp deletion | Duplication ~ 990 bp | Chr 7 |
| 2 | Normal | 120 bp deletion | Inversion of the Chromosome | Chr 1 |
| 3 | Normal | Normal | Duplication - 4 bp | Chr 1 |
| 4 | Normal | Normal | Deletion - 9 bp | Chr 1 |

**Supplementary Table 3: Raltegravir resistant mutant N155H**

|  | U-LTR | D-LTR | Chromosome | Chr # |
| --- | --- | --- | --- | --- |
| No Drug |  |  |  |  |
| 1 | 17 bp deletion | Normal | Duplication ~ 1.6Kb | Chr X |
| 2 | Normal | 2 bp deletion | Duplication ~ 1Kb | Chr 12 |
| 3 | 3 bp deletion | Normal | Duplication ~ 857 bp | Chr 1 |
| 4 | Normal | Entire LTR deleted | Inversion of the Chromosome | Chr 1 |
| 5 | Normal | 6 bp deletion | Duplication - 66 bp | Chr 19 |
|  |  |  |  |  |
| IC_50_ |  |  |  |  |
| 1 | Normal | Normal | Deletion – 31 bp | Chr 1 |
| 2 | Normal | 27 bp deletion | Deletion – 18 bp |  |
| 3 | Normal | Normal | Duplication ~ 1.7Kb | Chr 15 |
| 4 | Normal | Normal | Duplication ~ 820 bp | Chr 12 |
| 5 | Normal | Normal | Duplication ~ 3.7 Kb | Chr 12 |
| 6 | Normal | 3 bp deletion | Duplication - 200 bp | Chr 4 |
|  |  |  |  |  |
| IC_75_ |  |  |  |  |
| 1 | 10 bp deletion | Normal | Duplication ~ 1 Kb | Chr 2 |
| 2 | Normal | Normal | Duplication 4 bp | Chr 20 |
|  |  |  |  |  |
| PBMC No Drug | |  |  |  |
| 1 | 13 bp deletion | Normal | Duplication - 330 bp | Chr 1 |
| 2 | Normal | Normal | 1 bp deletion | Chr 7 |
| 3 | Normal | Normal | Insertion of DNA from another Chromosome | Chr 1 & 11 |
| 4 | Normal | Normal | Duplication - 36 bp | Chr 6 |
| 5 | Normal | Entire LTR deleted | Duplication ~ 840 bp | Chr 9 |
| 6 | 450 bp deletion | Normal | Inversion of the Chromosome | Chr 8 |
| 7 | Normal | Normal | Deletion - several Kb | Chr 17 |
| 8 | Normal | 8 bp normal | Inversion of the Chromosome | Chr 14 |
| 9 | 23 bp deletion | Normal | Duplication - 167 bp | Chr 12 |
| 10 | Entire LTR deleted | Normal | Circular viral DNA carrying 242 bp of host sequence between the proviral ends | Chr 13 |

**Supplementary Table 4: Raltegravir resistant mutant G140S/Q148H**

|  | U-LTR | D-LTR | Chromosome | Chr # |
| --- | --- | --- | --- | --- |
| No Drug |  |  |  |  |
| 1 | Normal | Normal | Duplication - 388 bp | Chr 9 |
| 2 | Normal | 3 bp insertion | Duplication - 5 bp | Chr 5 |
| 3 | 10 bp insertion | Normal | Inversion of chromosome | Chr 1 |
| 4 | Normal | Normal | Duplication - 152 bp | Chr 21 |
| 5 | Normal | 2 bp insertion | Duplication - 6 bp | Chr 2 |
|  |  |  |  |  |
| IC_50_ |  |  |  |  |
| 1 | 5 bp insertion | 1 bp (A) deletion | Chr 3 at D-LTR end. No match for 5bp adjacent to the provirus immediately followed by Bcl1 site and sequence from Chr 10 | Chr 3 & 10 |
| 2 | Full deletion | Normal | Duplication - 6 bp | Chr 14 |
| 3 | 32 bp insertion | Normal | Insertion of DNA from a different chromosome | Chr 3 & 13 |
| 4 | Normal | Normal | Duplication - 514 bp | Chr 17 |
| 5 | 1 bp insertion | 5 bp insertion | Duplication - 10 bp | Chr 18 |
|  |  |  |  |  |
| IC_75_ |  |  |  |  |
| 1 | 11 bp deletion | Normal | Duplication -~ 420 bp - Bcl1 site within the duplication | Chr 7 |
| 2 | Full deletion | 1 bp insertion | Insertion of DNA from a different chromosome | Chr 3 & 4 |
| 3 | Full deletion | 5 bp insertion | Duplication - > 600 bp Bcl1 site within duplication | Chr 1 |
| 4 | Normal | 3 bp deletion | Duplication - 95 bp | Chr 12 |
| 5 | Normal | Full deletion | Insertion of DNA from a different chromosome | Chr 10 & 21 |
| 6 | Full deletion | Normal | Duplication - 3bp | Chr 3 |
| 7 | 65 bp deletion | Full deletion | Insertion of DNA from a different chromosome | Chr 4, X & 11 |

**Table Legends:**

Structure of the aberrant proviruses isolated from cells treated with suboptimal concentrations of EVG (Table S1); from cells infected with viruses harboring RAL resistant mutations and treated with suboptimal doses of RAL (Tables S2-S4). The inhibitory concentrations of EVG or RAL used to treat the cells are indicated. The aberrations in the viral LTRs and in the integrated host chromosome(s) are indicated. U-LTR: the LTR adjacent to the primer binding site; D-LTR: the LTR adjacent to the polypurine tract.
